# The Interactive Effects of Affect Lability, Negative Urgency, and Sensation Seeking on Young Adult Problematic Drinking

**DOI:** 10.1155/2013/636854

**Published:** 2012-12-27

**Authors:** Kenny Karyadi, Ayca Coskunpinar, Allyson L. Dir, Melissa A. Cyders

**Affiliations:** Indiana University-Purdue University, Indianapolis, IN, USA

## Abstract

Prior studies have suggested that affect lability might reduce the risk for problematic drinking among sensation seekers by compensating for their deficiencies in emotional reactivity and among individuals high on negative urgency by disrupting stable negative emotions. Due to the high prevalence of college drinking, this study examined whether affect lability interacted with sensation seeking and negative urgency to influence college student problematic drinking. 414 college drinkers (mean age: 20, 77% female, and 74% Caucasian) from a US Midwestern University completed self-administered questionnaires online. Consistent with our hypotheses, our results indicated that the effects of sensation seeking and negative urgency on problematic drinking weakened at higher levels of affect lability. These findings emphasize the importance of considering specific emotional contexts in understanding how negative urgency and sensation seeking create risk for problematic drinking among college students. These findings might also help us better understand how to reduce problematic drinking among sensation seekers and individuals high on negative urgency.

## 1. Introduction 

 Young adult college students in the United States are at a heightened risk for alcohol use problems due to their hazardous patterns of alcohol use [1–4]. Particularly, among 14000 students from 119 universities, 31% endorsed criteria for alcohol abuse and 6% endorsed criteria for alcohol dependence [5]. However, few college students seek treatment for alcohol use problems [1, 5], suggesting a need to identify risk factors for problematic drinking among these students. The National Institute on Alcohol Abuse and Alcoholism (NIAAA) has stated that these young adults have personality traits and psychological vulnerabilities that place them at increased risk for problems with alcohol [6]. The present study examined three traits that have been associated with problematic drinking: negative urgency (tendency to behave impulsively in face of strong negative emotions), sensation seeking (tendency to pursue stimulation through impulsive behaviors), and affect lability (rapidly changing affective states) [7, 8]. 

 Sensation seekers are thought to use alcohol to attain stimulation [9], whereas individuals who are high on negative urgency might use alcohol to alleviate negative emotions [10], and affectively labile individuals might use alcohol to regulate affective fluctuations [7]. Even though these characteristics indicate different pathways for alcohol use, all three traits have been associated with problematic drinking. However, there are inconsistencies: although sensation seeking has been associated with problematic alcohol use cross-sectionally [11, 12] and prospectively [13], other studies have failed to find this association either cross-sectionally [10] or prospectively [14]. Similarly, negative urgency has been associated with problematic drinking [9, 15, 16], but some research has failed to find the negative urgency-problematic drinking association cross-sectionally [17, 18] and prospectively [14]. Finally, affectively labile individuals have also been shown to engage in problematic drinking [7, 19–21], but the affect lability-problematic drinking association is not always consistent [20, 22]. 

Taken together, these findings indicate that the effects of sensation seeking, negative urgency, and affect lability on problematic drinking are inconsistent. One potential explanation for these inconsistencies is the presence of an interactive effect. Indeed, prior findings indicated that affect lability interacted with broader impulsivity traits to influence problematic drinking [7, 23], supporting the possibility that affect lability might also interact with more specific forms of impulsivity. However, those findings indicated that high levels of affect lability strengthened the effects of impulsivity traits on problematic drinking. In contrast, the present study proposed that higher levels of affect lability will attenuate the effects of sensation seeking and negative urgency on problematic alcohol use. Specifically, we proposed that affect lability would compensate for emotional reactivity deficiencies among sensation seekers and would disrupt stable negative emotions among individuals high on negative urgency, both of which would reduce the risk for problematic drinking. 

It has been theorized that sensation seekers have deficiencies in emotional reactivity [24]. Indeed, prior studies indicated that sensation seekers are less reactive to aversive stimuli [25] and threatening images [26]. Sensation seekers might engage in risky behaviors, such as alcohol use [10], in order to achieve stimulation and to compensate for these deficiencies in emotional reactivity [24, 27]. At the same time, affect lability has been shown to be present in some sensation seekers [24] and has been characterized as enhanced emotional reactivity [28]. High levels of affect lability might compensate for deficiencies in emotional reactivity among sensation seekers. If this is the case, affectively labile sensation seekers may be less likely to use alcohol as a means of compensation. We hypothesized that high levels of affect lability would weaken the effect of sensation seeking on problematic drinking. 

 Individuals who are high on negative urgency have been thought to be emotionally dysregulated. The experience of negative emotions might cause these individuals to focus on their immediate emotional needs [29, 30] and to engage in risky behaviors, such as alcohol use, in order to address those emotional needs [10]. Furthermore, prior findings indicated that risky behaviors among individuals high on urgency are driven by strong and stable emotional states [31, 32], suggesting that problematic alcohol use among individuals high on negative urgency might also be driven by strong and stable negative emotions. In contrast, affect lability is characterized by fluctuations in affective states [7]. The presence of affect lability among individuals high on negative urgency might undermine the strong and stable negative emotions needed to drive alcohol use. We hypothesized that high levels of affect lability would weaken the effect of negative urgency on problematic drinking. 

## 2. Materials and Methods

### 2.1. Participants and Procedure

Study data were obtained from undergraduate students (*n* = 785) enrolled in lower level psychology courses at a US Midwestern University. The final study sample was restricted to ages 18–25, in order to focus on young adults, as recommended by NIAAA [33]. Furthermore, the final study sample was also restricted to those who consumed alcohol on at least a monthly basis (*n* = 414), in order to ensure that observed effects were not confounded by abstention. About 77% of the final sample was female and 23% was male. The mean age of the sample was 20.11 years old (SD = 1.79). The sample was comprised of about 74% European American, 9% African American, 5% Hispanic American, and 3% Asian American—with the remaining 9% comprising other races. The original and final samples did not significantly differ on race or sex. The study was approved by the university's Institutional Review Board. Participants completed the study in one session using a web-based questionnaire and were awarded course credit for participation.

### 2.2. Measures (See Table 1)

#### 2.2.1. Hazardous Alcohol Use and Alcohol-Related Problems

We decided to use two subscales of the Alcohol Use Disorder Identification Test rather than the full scale [34]. This is because the subscales allow us to measure specific constructs underlying problematic alcohol use, including alcohol-related problems and hazardous patterns of drinking, whereas the full scale assesses the risk for alcohol use disorders. Additionally, as suggested by Coskunpinar and colleagues [35], disaggregating alcohol outcomes will lead to more robust prediction by impulsivity-related traits. All items were rated on a 5-point Likert scale, with higher ratings indicating higher levels of alcohol involvement. The hazardous alcohol use subscale consists of 2 items (*α* = .83) and was calculated as a sum, with higher summed values indicating greater levels of hazardous alcohol use. This subscale assesses frequency of heavy drinking and typical quantity of drinking. Alcohol-related problems consist of 4 items (*α* = .67), with higher summed values indicating greater levels of alcohol-related problems. This subscale assesses whether participants have ever felt guilty after drinking, had blackouts and other alcohol-related injuries, and have had others expressed concerns about their drinking. 

#### 2.2.2. Sensation Seeking and Negative Urgency

Sensation seeking and negative urgency were assessed using subscales of the UPPS-P Impulsive Behavior Scale—which is a 59-item inventory designed to measure personality pathways to impulsive behavior [36]. All items were assessed in terms of likelihood of occurrence, with response options ranging from (1) “Disagree Strongly” to (4) “Agree Strongly.” The subscales were calculated as separate means, with higher mean values indicating higher levels of the trait. The sensation seeking subscale consists of 12 items, which assess the tendency to seek out stimulation and excitement (*α* = .87). The negative urgency subscale consists of 12 items, which assess impulsive behaviors that are related to negative affect (*α* = .86). 

#### 2.2.3. Affect Lability

Affect lability was assessed using the Affective Lability Scale-Short Form, which is an 18-item scale designed to measure fluctuations in affective states [37]. The ALS-SF has three subscales, which include anxiety and depression lability, anger lability, and depression and elation lability. The anxiety-depression lability subscale consists of 5 items, which assess fluctuations between anxiety and depression (*α* = .90). The anger lability subscale consists of 5 items, which assess changes in affective states from neutral to anger (*α* = .89). The depression-elation lability subscale consists of 8 items, which assess fluctuations between depression and elation (*α* = .88). Response options for these items ranged from (1) “Very characteristic of me” to (4) “Very uncharacteristic of me.” Separate mean values were calculated for each subscale, with higher mean values indicative of higher degrees of affect lability. 

### 2.3. Analytic Strategy

Using SPSS 19.0, we examined bivariate correlations among all study variables and performed a series of multiple regression and simple slope analyses. All continuous predictors were centered to facilitate interpretation of the interaction coefficients, and significant interactions were probed at the mean and +1/−1 SD of the moderator using simple slope analyses [38]. Because problematic alcohol use has been shown to differ between men and women [39], gender was included as a covariate in all analyses. Age has also been differentially associated with problematic alcohol use [40] and was included as a covariate. We tested all potential covariates by predictor interactions to ensure that the effects of the predictors were independent of the covariates and considered retaining any interactions that were significant at *P* < .01 to guard against alpha inflation. No covariate by predictor interactions met this criterion, suggesting that the effects of the covariates were independent of those of the predictors. 

## 3. Results

We first examined the correlations among our predictors and outcomes. Sensation seeking (*r* = .13, *P* < .01) and negative urgency (*r* = .28, *P* < .01) were both positively correlated with alcohol-related problems. Similarly, negative urgency (*r* = .21, *P* < .01) and sensation seeking (*r* = .26, *P* < .01) were positively correlated with hazardous alcohol use. Anxiety-depression lability (*r* = .13, *P* < .01), depression-elation lability (*r* = .16, *P* < .01), and anger lability (*r* = .16, *P* < .01) were positively correlated with alcohol-related problems, but not with hazardous alcohol use. Negative urgency, but not sensation seeking, was positively correlated with all the affect lability scales, with correlations ranging from .39 to .51 (all *P* < .01).  Table 2 provides correlations among the predictors and outcomes. 

**Figure 1:**
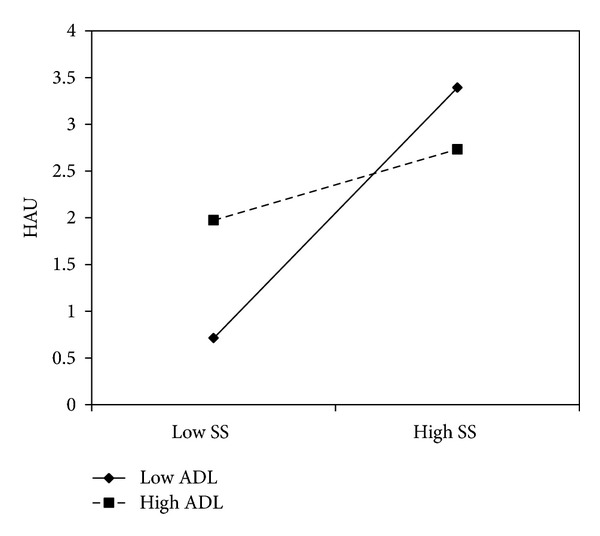
Anxiety and depression lability moderated the effect of sensation seeking on hazardous alcohol use among college students.

**Figure 2:**
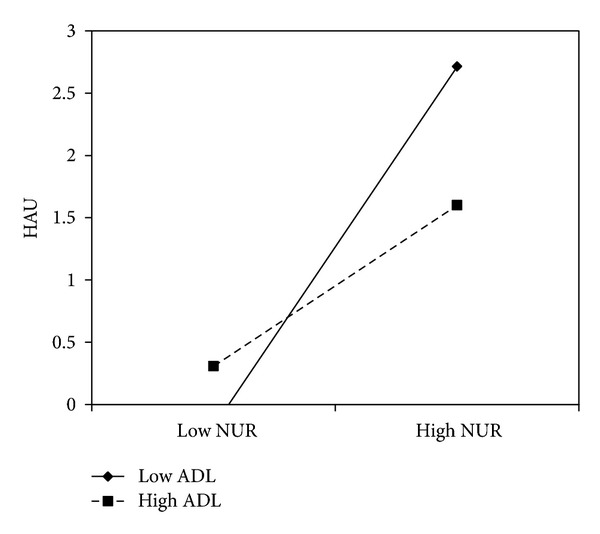
Anxiety and depression lability moderated the effect of negative urgency on hazardous alcohol use among college students.

 Next, we tested whether the specific affect lability traits moderated the effects of sensation seeking and negative urgency on alcohol-related problems and hazardous alcohol use. For hazardous alcohol use, the effect of sensation seeking was moderated by anxiety-depression lability (*b* = −.48, *P* = .02). In this analysis, both gender (*b* = 1.17, *P* < 0.0001) and age (*b* = 0.15, *P* = 0.02) had significant effects on hazardous alcohol use. Simple slope analyses indicated that sensation seeking was associated with hazardous alcohol use at low levels of anxiety-depression lability (*b* = 1.82, *P* < .001), but this effect weakened for those at mean anxiety-depression lability (*b* = 0.86, *P* < .001) and was nonsignificant at high levels of anxiety-depression lability (*b* = −0.09, *P* = 0.85) (see Figure 1). Furthermore, the interaction between negative urgency and anxiety-depression lability on hazardous alcohol use approached significance (*b* = −.40, *P* = .06). Both gender (*b* = 1.29, *P* < 0.0001) and age (*b* = 0.20, *P* = 0.002) had significant effects on hazardous alcohol use. The effects of negative urgency on hazardous alcohol use were positive and significant among those low on anxiety-depression lability (*b* = 1.84, *P* < .001) and at mean anxiety-depression lability (*b* = 1.05, *P* < .001), but not at high levels of anxiety-depression lability (*b* = 0.25, *P* = 0.60) (see Figure 2). 

**Figure 3:**
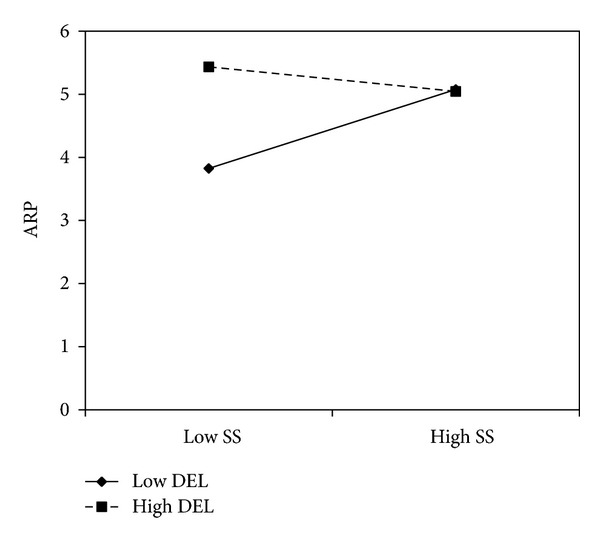
Depression and elation lability moderated the effect of sensation seeking on alcohol-related problems among college students.

**Figure 4:**
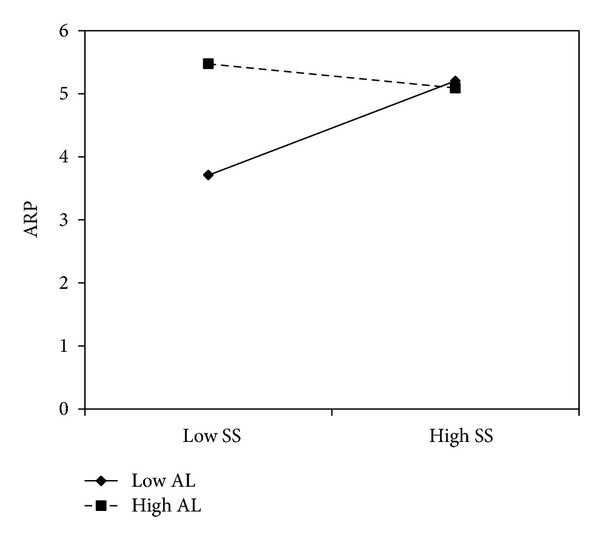
Anger lability moderated the effect of sensation seeking on alcohol-related problems among college students.

For alcohol-related problems, the effect of sensation seeking was moderated by anger lability (*b* = −.41, *P* = .01). Gender (*b* = 0.49, *P* = 0.03), but not age (*b* = −0.01, *P* = 0.89), had a significant effect on alcohol-related problems. Simple slope analyses indicated that sensation seeking was associated with alcohol-related problems at low levels of depression-elation lability (*b* = 1.10, *P* = .02), but the effect weakened at mean (*b* = 0.22, *P* = 0.16) and high levels of depression-elation lability (*b* = −0.66, *P* = 0.18) (see Figure 3). Furthermore, the interaction between depression-elation lability and sensation seeking approached significance (*b* = −.41, *P* = .05). Once again, gender had a significant effect on alcohol-related problems (*b* = 0.47, *P* = 0.04), but age did not (*b* = −0.002, *P* = 0.97). Sensation seeking was associated with alcohol-related problems at low levels of anger lability (*b* = 1.14, *P* = .002), but not at mean (*b* = 0.28, *P* = 0.07) and high levels of anger lability (*b* = −0.59, *P* = 0.12) (see Figure 4).  Table 3 summarizes the interaction results. 

## 4. Discussion

 Consistent with our hypotheses, our results indicated that sensation seekers and individuals high on negative urgency are at lower risk for hazardous alcohol use and alcohol-related problems, but only within the context of higher affect lability traits. These findings help explain prior inconsistencies in the literature [11, 14, 16] by emphasizing the importance of considering specific emotional experiences in understanding how negative urgency and sensation seeking create risk for problematic drinking. Moreover, these findings also clarify prior impulsivity-affect lability interactions [7, 23] by showing that affect lability interacts with more specific forms of impulsivity differently. Finally, these findings emphasize the importance of considering these three factors when addressing problematic drinking among college students. 

 It has been theorized that sensation seekers engage in risky behaviors, such as alcohol use [10], to compensate for deficiencies in emotional reactivity [24, 41]. Our findings provide some support for the notion that the concurrent experience of affect lability might compensate for these deficiencies. Specifically, college students who are high on both sensation seeking and affect lability might have less emotional reactivity deficiencies and might consequently be less likely to use alcohol to compensate for emotional reactivity deficiencies and to experience problems from alcohol use. Relatedly, although affect lability is not typically considered a protective factor, these findings suggest that problematic drinking among sensation seeking college students could be reduced by increasing emotional reactivity. However, future studies are needed to examine the extent to which emotional reactivity might protect sensation seeking college students from engaging in problematic drinking. 

 Furthermore, our results support prior findings indicating that the urgency-risky behavior association is strengthened by strong and stable emotions [31, 32] by showing that affective fluctuations weaken the effect of negative urgency on problematic drinking. Specifically, when college students high on negative urgency experience affective fluctuations, their current emotional states might not be stable enough to drive alcohol use behaviors. Again, although affect lability is not a protective factor, these findings indicate that one approach of reducing problematic drinking among college students high on urgency is by disrupting strong and stable emotions. Indeed, prior studies have indicated multiple therapeutic approaches that are effective for dealing with strong distressing emotions and alcohol use [42]. However, future studies are needed to examine whether disrupting strong and stable negative emotions among college students high on urgency directly lead to a reduction in problematic drinking behaviors. 

 Finally, we have thus far discussed affect lability as a protective factor among college students who are high on urgency and sensation seeking. However, prior studies have indicated that affectively labile individuals engage in problematic drinking as a way of coping with affective fluctuations [7, 19–21]. Based on these prior studies, affect lability does not appear to be a protective factor. At the same time, there are some features of affect lability that might confer protection against problematic drinking for certain individuals. For instance, affect lability might render sensation seeking college students more emotionally reactive and might disrupt stable negative emotions among college students high on negative urgency. Future studies should examine whether cultivating these specific features of affect lability can reduce problematic drinking among college students high on negative urgency and sensation seeking. 

The current study does have some limitations, which may hamper its generalizability. First, the cross-sectional nature limits causal inferences. Second, the sample was comprised of mostly Caucasian, female, young adults. Although this sample of young adults helps attain the goal of understanding problematic alcohol use among young adults given their increased risk, it is unclear how the current results would generalize to other more diverse sample. Furthermore, the internal consistency coefficient for our measure of alcohol-related problems was low (*α* = .67), which may have limited our power to detect the effects of our predictors on alcohol-related problems. Additionally, the current study did not examine how affect lability might interact with other impulsivity-related traits, such as positive urgency. Finally, the interaction effects were small (*b* < 3), possibly limiting the clinical relevance of our findings and indicating that future studies should replicate these findings using college students with alcohol use disorders in order to further support clinical implications. 

## 5. Conclusions

 Overall, these results indicated that affect lability seems to alter the effects of sensation seeking and negative urgency on problematic drinking. These results suggest that we must take into account emotional stability and reactivity in order to elucidate whether sensation seeking and negative urgency are creating risk for problematic alcohol use among college students. Our results also provide some support for the development of alcohol interventions that focus on disrupting stable negative emotions and that focus on increasing emotional reactivity. Although affect lability is not necessarily a protective factor, practitioners and researchers should still consider how increasing some features of affect lability might be beneficial for college drinkers who are high on negative urgency and sensation seeking.

## Figures and Tables

**Table 1:** Measures information.

Measures	Male		Female		Total		*t*-test
	M	SD	M	SD	M	SD	P
Sensation seeking	3.04	0.56	2.77	0.60	2.83	0.60	<0.0001
Negative urgency	2.35	0.57	2.41	0.62	2.39	0.61	0.44
Anxiety-depression	1.73	0.82	2.07	0.84	1.99	0.84	0.001
Depression-elation	2.07	0.69	2.17	0.69	2.15	0.69	0.25
Anger	1.73	0.76	1.88	0.81	1.84	0.80	0.10
Hazardous drinking	7.85	2.94	6.34	2.09	6.69	2.39	<0.0001
Alcohol problems	5.86	2.33	5.27	1.67	5.41	1.86	0.007

Note: Independent samples *t*-test were conducted to examine whether scale scores differ between men and women.

**Table 2:** Correlations among predictors and outcomes.

Variables	1	2	3	4	5	6	7
(1) HAU	1	.61**	.26**	.21**	.001	.02	.06
(2) ARP		1	.13**	.28**	.13**	.16**	.16**
(3) SS			1	.08	−.09	.07	.04
(4) NUR				1	.45**	.40**	.51**
(5) ADL					1	.68**	.69**
(6) DEL						1	.63**
(7) AL							1

Note: *indicates *P* < 0.05, **indicates *P* < 0.01. HAU: hazardous alcohol use, ARP: alcohol-related problems, SS: sensation seeking, NUR: negative urgency, ADL: anxiety-depression lability, DEL: depression-elation lability, and AL: anger lability.

**Table 3:** Interactions among sensation seeking, negative urgency, and affect lability on hazardous alcohol use and alcohol-related problems.

Predictors	HAU					ARP				
	*b *	SE	**β**	Δ*R* ^2^	*P *	*b *	SE	**β**	Δ*R* ^2^	*P *
SS × ADL	−**.48**	**.20**	**−.11**	**.01**	**.02**	−.29	.17	−.09	.01	.08
										
SS × DEL	−.47	.27	−.09	.01	.08	**−.41**	**.21**	**−.10**	**.01**	**.05**
										
SS × AL	−.19	.23	−.04	.00	.41	**−.47**	**.19**	**−.12**	**.02**	**.01**
										
NUR × ADL	**−.40**	**.22**	**−.09**	**.01**	**.06**	−.06	.17	−.02	.00	.73
										
NUR × DEL	−.16	.26	−.03	.00	.55	−.02	.21	−.01	.00	.91
										
NUR × AL	−.30	.23	−.07	.00	.18	−.18	.18	−.05	.00	.33

Note: bolded coefficients were significant at *P* < .05, and Δ*R* ^2^ refers to change in *R* ^2^ in the third step of the analyses (when the interaction term was entered). HAU: hazardous alcohol use, ARP: alcohol-related problems, SS: sensation seeking, NUR: negative urgency, ADL: anxiety-depression lability, DEL: depression-elation lability, and AL: anger lability.
